# Multistep coevolution of HIV-1 and human leukocyte antigen-C-restricted HIV-1-specific CD8^+^ T cells and the association with disease progression

**DOI:** 10.1093/pnasnexus/pgag105

**Published:** 2026-04-07

**Authors:** Takayuki Chikata, Tomohiro Akahoshi, Yu Zhang, Hung The Nguyen, Shinichi Oka, Hiroyuki Gatanaga, Masafumi Takiguchi

**Affiliations:** Division of International Collaboration Research and Tokyo Laboratory, Joint Research Center for Human Retrovirus Infection, Kumamoto University, 2-2-1 Honjo, Chuo-ku, Kumamoto 860-0811, Japan; AIDS Clinical Center, National Center for Global Health and Medicine, Japan Institute for Health Security, Tokyo 162-8655, Japan; Division of International Collaboration Research and Tokyo Laboratory, Joint Research Center for Human Retrovirus Infection, Kumamoto University, 2-2-1 Honjo, Chuo-ku, Kumamoto 860-0811, Japan; Division of International Collaboration Research and Tokyo Laboratory, Joint Research Center for Human Retrovirus Infection, Kumamoto University, 2-2-1 Honjo, Chuo-ku, Kumamoto 860-0811, Japan; Division of International Collaboration Research and Tokyo Laboratory, Joint Research Center for Human Retrovirus Infection, Kumamoto University, 2-2-1 Honjo, Chuo-ku, Kumamoto 860-0811, Japan; AIDS Clinical Center, National Center for Global Health and Medicine, Japan Institute for Health Security, Tokyo 162-8655, Japan; AIDS Clinical Center, National Center for Global Health and Medicine, Japan Institute for Health Security, Tokyo 162-8655, Japan; Division of International Collaboration Research and Tokyo Laboratory, Joint Research Center for Human Retrovirus Infection, Kumamoto University, 2-2-1 Honjo, Chuo-ku, Kumamoto 860-0811, Japan

**Keywords:** HIV-1, T cell, HLA-C, coevolution, mutation

## Abstract

Disease progression during HIV-1 infection is proposed to correlate with the accumulation of virus variants that have evaded HIV-1-specific T cells; however, the multistep coevolution of HIV-1 with HIV-1-specific T cells has not been well investigated. Here, we analyzed the coevolution of HIV-1 with human leukocyte antigen (HLA)-C*14-restricted HIV-1-specific T cells in treatment-naive HLA-C*14^+^ people living with HIV-1 (PLWH). The analyses of T cells specific for NefYT9 wild-type (WT) or escape mutant epitopes and HIV-1 sequences in HLA-C*14^+^ PLWH suggested that NefQ125H was first selected by NefYT9 WT–specific T cells, then NefQ125D was selected by NefYT9-6H-specific T cells. This coevolution was confirmed by longitudinal analysis of HLA-C*14^+^ PLWH. The CD4 count in NefQ125D virus–infected HLA-C*14^+^ individuals was significantly lower than that in WT-virus–infected HLA-C*14^+^ individuals, suggesting that accumulation of the NefQ125D mutant epitope correlates with disease progression. Our findings demonstrate that the multistep coevolution of HIV-1 with HLA-C-restricted HIV-1-specific T cells is associated with disease progression in HIV-1 infection.

Significance statement The impact on HIV-1 disease progression by the emergence of escape mutations selected by HIV-1-specific human leukocyte antigen (HLA)-A/B-restricted T cells and coevolution of HIV-1 with these HLA-A/B-restricted T cells was well known, whereas the coevolution with HLA-C-restricted T cells is not well investigated. We show the five-step coevolution of HIV-1 with HLA-C*14-restricted T cells specific for NefYT9 or its mutant epitopes and a significant correlation between the accumulation of secondary escape mutation, NefQ125D, and lower CD4 T-cell count in HLA-C*14^+^ people living with HIV-1. The present study demonstrates that HLA-C-restricted T cells are involved in the coevolution of HIV-1 with HIV-1-specific T cells.

## Introduction

HIV-1 harboring certain mutations can evade the killing activity of HIV-1-specific cytotoxic T lymphocytes (CTLs). These escape mutations affect the recognition of HIV-1-specific T-cell receptor (TCR), epitope peptide binding to human leukocyte antigen (HLA) molecules, and intracellular antigen processing (1–11). Accumulation of CTL escape mutations at the population level was assumed indirectly using comprehensive sequencing-based analysis of HLA-associated polymorphisms (APs) in the virus sequence (12–21). The number of HLA-B-APs was higher than that of HLA-A-APs or HLA-C-APs (13–15, 18, 21, 22), suggesting that HIV-1-specific HLA-B-restricted CD8^+^ T cells contribute more significantly to the selection of escape mutations than HLA-A-restricted or HLA-C-restricted CD8^+^ T cells. A small number of reported HLA-APs have been demonstrated to be escape mutations based on the fact that these substitutions affect the recognition of HIV-1-specific CD8^+^ T cells (7, 9, 10, 12, 21), with these escape mutations mainly residing within HLA-A-restricted or HLA-B-restricted epitopes. However, it remains unclear how HLA-C-restricted CTLs contribute to selecting escape mutations and suppressing the replication of HIV-1 circulating in people living with HIV-1 (PLWH).

The expression levels of the HLA-C molecule are lower than those of HLA-A and HLA-B molecules on normal cells (23–25) and on HIV-1-infected cells (23–25); therefore, the number of epitope peptides presented by HLA-C on the cell surface is relatively lower than those presented by HLA-A or HLA-B. A previous study demonstrated that the expression level of each HLA-C allele inversely correlated with the HIV-1 plasma viral load (pVL) in treatment-naive PLWH, and there was a strong positive correlation between the expression level of HLA-C alleles and HIV-specific T-cell responses (26). This study suggested that HLA-C-restricted T cells exert a strong immune pressure on HIV-1 if the surface expression level of HLA-C allele is high. Previous studies showed that HLA-C*12:02-restricted T cells specific for two epitopes effectively suppressed HIV-1 replication in treatment-naive PLWH (27) and PolT290L, GagV280T, PolS653A/T/L, and EnvF383L mutations were selected by HLA-C*12:02, HLA-C*01:02, HLA-C*15:05, and HLA-Cw4-restricted CD8^+^ T cells, respectively (28–30). These findings suggest that some HLA-C-restricted T cells have the ability to suppress HIV-1 replication and to select HIV-1 escape mutants. However, as only a small number of studies investigating HIV-1 control or selection of HIV-1 escape mutants by HLA-C-restricted T cells have been reported (27–31), it remains unclear to what extent HLA-C-restricted T cells contribute to selecting HIV-1 escape mutants and controlling HIV-1 disease progression.

Certain escape mutations generate new T-cell epitopes that induce mutant-specific T cells only if these mutant epitope peptides can bind to HLA class I (28, 32–36). However, these mutant-specific T cells did not select additional mutations in most cases. The selection of a second mutation in the same epitope or at the same position by T cells specific for the first mutation was found in only two cases. HLA-B*27-restricted GagKK10-specific T cells (GagKK10: KRWIILGLNK_263–272_) selected GagL268M mutant virus and then HLA-B*27-restricted T cells cross-reacting to GagKK10 and GagKK10-6M peptides were elicited (8, 37). The cross-reacting T cells then selected GagR264K mutant virus, whereas the GagKK10-2K mutant-specific T cells were not elicited in HLA-B*27^+^ individuals infected with GagR264K virus (37). In this case, two mutations were accumulated at different positions (Gag264 and Gag268) in the GagKK10 epitope. Another example is the HLA-B*51:01/HLA-B*52:01-restricted epitope, PolTI8 (RT128-135: TAFTIPSI) (38). RTI135V virus was selected by PolTI8-specific HLA-B*52:01-restricted T cells in individuals harboring an HLA-B*52:01/C*12:02 haplotype. As a new epitope, PolTN9-8V (RT128-136: TAFTIPSVN) was presented by HLA-C*12:02, and HLA-C*12:02-restricted PolTN9-8V-specific T cells were elicited in RTI135V virus–infected individuals harboring this haplotype. HLA-C*12:02-restricted PolTN9-8V-specific T cells further selected RTI135L mutant virus. Thus, as the multistep coevolution of HIV-1 with HIV-1-specific T cells has only been analyzed in a few cases, further research is required.

A previous study demonstrated the highest expression of HLA-C*14:02 on the cell surface among 17 HLA-C alleles (26), suggesting that this allele may effectively elicit HIV-1-specific T cells. Indeed, CD8^+^ T cells specific for six novel HLA-C*14-restricted HIV-1 epitopes were identified in a previous study using immunopeptidomics (39). Our comprehensive analysis of HLA-APs in an HIV-1-infected Japanese cohort revealed an adapted HLA-C*14:02-AP and/or HLA-C*14:03-AP (HLA-C*14:02/C*14:03-AP) at positions 1 and 6 in the NefYT9 epitope (Nef120-128: **Y**FPDW**Q**NYT), an adapted HLA-C*14:02/C*14:03-AP at position 7 in NefIF8 (Nef114-121: IYHTQG**Y**F), and an adapted HLA-C*14:02/C*14:03-AP at position 4 in GagLY9 (Gag78-86: LYN**T**IAVLY), whereas no HLA-C*14-AP was found in the other three epitopes (39). These studies suggest that escape mutant viruses carrying these HLA-C*14-APs are selected by T cells specific for these three epitopes and accumulate in the population. In the present study, we analyzed the coevolution of HIV-1 with HLA-C*14-restricted T cells. We found multistep coevolution of HIV-1 with HLA-C*14-restricted NefYT9-specific T cells and clarified the characteristics of the T cells elicited during the coevolution in HLA-C*14^+^ PLWH. Based on our findings, we suggest that the accumulation of virus harboring the second mutant may contribute to disease progression.

## Results

### HLA-APs in the HLA-C*14:02/03 epitope

We previously identified adapted HLA-C*14:02/HLA-C*14:03-APs in three epitopes, NefIF8, NefYT9, and GagLY9 (18). However, the statistical power may have been insufficient to detect robust HLA-APs because of the relatively small number of HLA-C*14:02^+^ or HLA-C*14:03^+^ treatment-naive PLWH analyzed in this cohort. HLA-C*14:02 and HLA-C*14:03 differed by only a single substitution at position 21. This position is located outside of the peptide binding groove, and it is therefore unlikely that the substitution at this position was directly involved in peptide binding to the HLA molecule or TCR recognition. Therefore, we combined individuals harboring both HLA-C*14 subtypes and reanalyzed the HLA-C*14-APs in the current study. We analyzed the association between the presence of HLA-C*14 (both HLA-C*14:02 and HLA-C*14:03) and HIV-1 amino acid substitutions in these three epitopes in 329 treatment-naive PLWH, including 23 newly identified and 306 previously identified individuals who were infected with subtype B (18). NefY120F, NefQ125D, NefQ125H, and GagT81A were significantly associated with the presence of HLA-C*14 (*P* = 1.6 × 10^−5^, *P* = 5.3 × 10^−3^, *P* = 1.6 × 10^−6^, and *P* = 8.1 × 10^−6^, respectively; Table S1). These results suggested that mutant viruses harboring these escape mutations may be selected by HLA-C*14-restricted T cell–mediated immune pressure. To investigate the multistep coevolution of HIV-1 with HLA-C-restricted CD8^+^ T cells, we focused on the accumulation of two mutations at Nef125 that are selected by HLA-C*14-restricted CD8^+^ T cells.

### Evolution of HIV-1 Nef125

**Figure 1:**
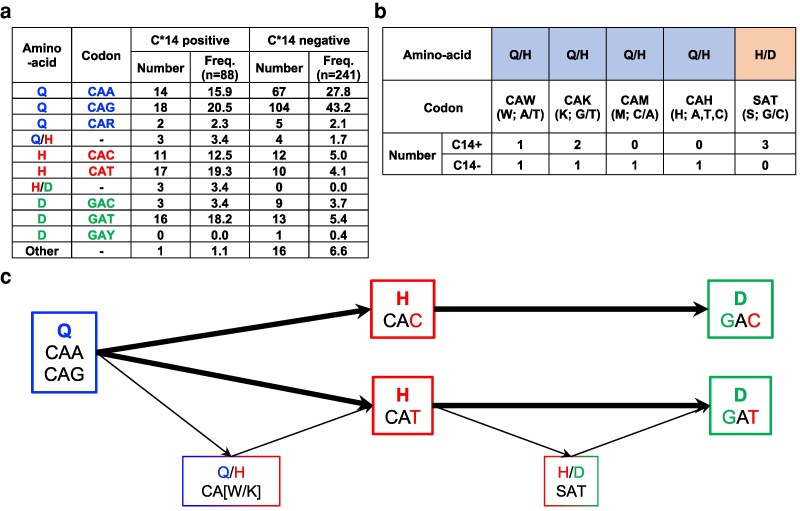
Amino acid and nucleotide substitutions at position 6 of NefYT9. a) Amino acid sequences and their corresponding codons in Nef125 as previously identified in 306 treatment-naive Japanese PLWH (18) and newly identified in 23 treatment-naive Japanese PLWH. b) Nucleotide sequence at Nef125 in 10 treatment-naive PLWH individuals for whom a mixture of amino acid sequences at Nef125 were detected in 329 treatment-naive Japanese PLWH. c) The hypothetical flow of nucleotide substitutions in the codon of Nef125.

We next analyzed the nucleotide sequences at Nef125 from 329 treatment-naive PLWH (88 HLA-C*14-positive and 241 HLA-C*14-negative individuals). Dominant amino acid sequences at Nef125 among these individuals were Gln, His, and Asp. Nucleotide sequences for Gln, His, and Asp were CAA/CAG, CAT/CAC, and GAT/GAC, respectively (Figure 1a). These findings imply two pathways of mutation: from CAA/CAG to GAT via CAT and from CAA/CAG to GAC via CAC. Mixtures of sequences of Gln and His (Q/H) and of His and Asp (H/D) were detected in 10 individuals (Figure 1b). In HLA-C*14^+^ PLWH, the Q/H mixture included two types of nucleotide sequence (CAW and CAK), whereas only one type of nucleotide sequence (SAT) was found in the H/D mixture (Figure 1b). These mixtures are located at intermediate positions between Nef125Q and NefQ125H or between NefQ125H and NefQ125D (Figure 1c). Taken together, these findings imply that the order of mutant selection at this position is from WT to Q125H, and then Q125H to Q125D.

### Induction and specificity of NefYT9 mutant–specific CD8^+^ T cells in individuals infected with Nef125 mutant virus

**Figure 2:**
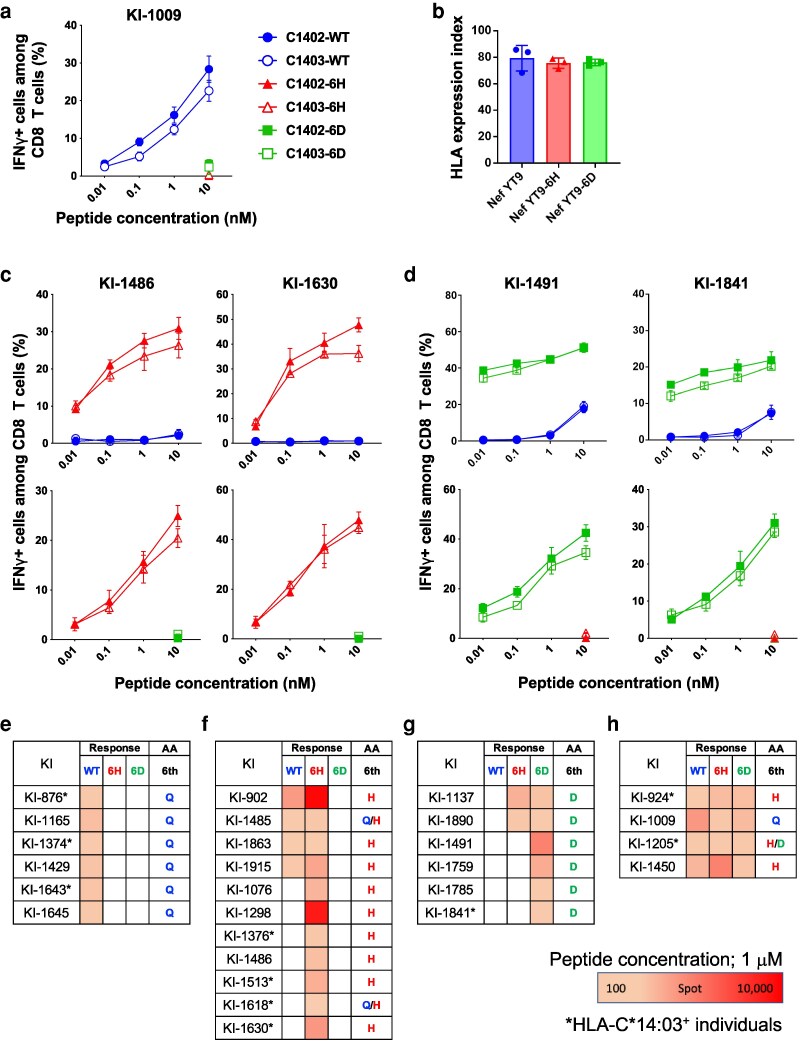
Induction and specificity of NefYT9-WT–specific and mutant-specific HLA-C*14-restricted CD8^+^ T cells. a) Recognition of NefYT9-WT (WT), NefYT9-6H (6H), and NefYT9-6D (6D) mutant peptides by NefYT9-specific T cells lines. IFN-γ production of NefYT9-reactive T-cell lines stimulated with CD4.221-C*14:02 or CD4.221-C*14:03 cells prepulsed with NefYT9 (WT), NefYT9-6H (6H), or Nef YT9-6D (6D) peptide was tested in ICS assays (*n* = 3). b) Binding ability of WT and two mutant epitope peptides at 100 μM to HLA-C*14:02. Binding affinity of these peptides to HLA-C*14:02 was measured by an HLA class I stabilization assay using the RMA-S-C*14:02 cell line. c) Recognition of NefYT9-WT and mutant peptides by NefYT9-6H-bulk T cells. IFN-γ production of NefYT9-6H-reactive T cells from KI-1486 (HLA-C*14:02^+^) and KI-1630 (HLA-C*14:03^+^) stimulated with CD4.221-C*14:02 or CD4.221-C*14:03 cells prepulsed with NefYT9-WT (WT), NefYT9-6D (6D), or NefYT9-6H (6H) was tested in ICS assays (*n* = 3). d) Recognition of NefYT9 and mutant peptides by NefYT9-6D-bulk CD8^+^ T cells. IFN-γ production of NefYT9-6D-bulk T cells from KI-1491 (HLA-C*14:02^+^) and KI-1841 (HLA-C*14:03^+^) stimulated with CD4.221-C*14:02 or CD4.221-C*14:03 cells prepulsed with NefYT9-WT (WT), NefYT9-6D (6D), or NefYT9-6H (6H) peptide were tested in ICS assays (*n* = 3). e–h) T-cell responses to NefYT9-WT (WT), NefYT9-6H (6H), or Nef YT9-6D (6D) peptides in 27 of 39 treatment-naive HLA-C*12:02^+^ PLWH were analyzed by an ex vivo ELISpot assay. Responders to Nef YT9-WT peptide only (E), NefYT9-6H peptide only, or both NefYT9-WT and NefYT9-6H peptides (F), NefYT9-D peptide only, or both NefYT9-6H and NefYT9-D peptides (G), and to all three peptides (H). The responses to each peptide at 1 µM were analyzed by an ex vivo ELISpot assay. The cutoff for a positive response was >100 spots/million PBMCs. Right column of each table shows the amino acid (AA) residue at position 6 of NefYT9 that was analyzed by direct-bulk sequencing. Asterisk indicates HLA-C*14:03-positive individuals. Plasma samples for sequence analysis were collected on the same day as PBMC samples for ex vivo ELISpot assays.

To confirm whether these two HLA-C*14-APs were selected by HLA-C*14-restricted NefYT9-specific T cell–mediated immune pressure, we established T-cell lines specific for NefYT9 wild type (WT) by stimulating peripheral blood mononuclear cells (PBMCs) from an HLA-C*14:02^+^ PLWH, designated KI-1009, with NefYT9-WT peptide. The T-cell lines were then tested for their ability to recognize 721.221 cells expressing HLA-C*14:02 (CD4.221-C*14:02 cells) or HLA-C*14:03 (CD4.221-C*14:03 cells) pulsed with NefYT9-WT, NefYT9-6H mutant, or NeFYT9-6D mutant peptide by an intracellular cytokine staining (ICS) assay. These NefYT9-specific T cells did not recognize the NefYT9-6D or NefYT9-6H mutant peptides (Figure 2a). This finding implied that NefQ125H and NefQ125D were selected as escape mutations by NefYT9-specific T cells.

Next, we investigated the binding of these mutant and WT epitope peptides to HLA-C*14:02 using the stabilization assay of HLA-C*14:02. The two mutant peptides bound effectively to HLA-C*14:02, and there was no significant difference in the binding ability of these three peptides (Figure 2b). To clarify whether T cells specific for NefYT9-6D or NefYT9-6H were elicited in HLA-C*14^+^ PLWH infected with these mutant viruses, we stimulated PBMCs from NefQ125D virus–infected HLA-C*14^+^ individuals (KI-1491;HLA-C*14:02 and KI-1841;HLA-C*14:03) with NefYT9-6D peptide and those from NefQ125H virus–infected HLA-C*14^+^ individuals (KI-1486:HLA-C*14:02 and KI-1630:HLA-C*14:03) with NefYT9-6H peptide, and cultured the cells for 2 weeks. The results of an ICS assay of these cultured bulk T cells demonstrated the induction of NefYT9-6H-specific T cells (Figure 2c) and NefYT9-6D-specific T cells (Figure 2d). These mutant-specific T cells did not cross-recognize the other mutant peptide (Figure 2c and d). Thus, NefYT9-6H-specific and NefYT9-6D-specific T cells were elicited in HLA-C*14^+^ individuals infected with NefQ125H and those infected with NefQ125D mutant virus, respectively.

Next, we investigated the frequency of T-cell responses to NefYT9-WT, NefYT9-6H, or NefYT9-6D peptide in 39 treatment-naive HLA-C*14^+^ PLWH using an ex vivo ELISpot assay. T-cell responses to at least one peptide were detected in 27 individuals. T-cell responses to NefYT9, NefYT9-6H, and NefYT9-6D were found in 14, 17, and 10 individuals, respectively (Figure 2e–h). Bulk sequence analysis of these 27 responders showed that WT, NefQ125H, and NefQ125D sequences were present in 7, 11, and 6 individuals, respectively, while mixtures of WT and NefQ125H and of NefQ125H and NefQ125D were detected in 2 and 1 individuals, respectively (Figure 2e–h). All six responders to WT peptide only were infected with WT virus (Figure 2e). Seven responders to 6H peptide only and four responders to both WT and 6H peptides were infected with NefQ125H virus or a WT/Q125H virus mixture (Figure 2f). Four responders to 6D peptide only and two responders to both 6H and 6D peptides were infected with NefQ125D virus (Figure 2g). These findings led to the hypothesis of multistep coevolution between NefYT9-specific T cells and HIV-1.

### HLA-C*14-tetramer analysis of T cells specific for NefYT9-WT and/or mutant epitopes in responders to two or all three NefYT9 epitope peptides

It is technically challenging to identify and determine the precise specificity of a small number of T cells by ex vivo ELISpot assays. We therefore attempted to identify CD8^+^ T cells specific for NefYT9 or its mutant epitopes using HLA-C*14:02-NefYT9, HLA-C*14:02-NefYT9-6H, and HLA-C*14:02-NefYT9-6D tetramers (WT-tet, 6H-tet, and 6D-tet, respectively). To evaluate the specificity of these tetramers, we analyzed PBMCs from KI-1643, KI-1298, and KI-1491 in whom T-cell responses to the single peptides NefYT9, NefYT9-6H, and NefYT9-6D were found, respectively (Figure 2e–g). We detected WT-tet binding (WT-tet^+^), 6H-tet binding (6H-tet^+^), and 6D-tet binding (6D-tet^+^) CD8^+^ T cells in KI-1643, KI-1298, and KI-1491, respectively, but two or three tetramer-binding CD8^+^ T cells were not detected (Figure S1A). These results demonstrated the high specificity of these three tetramers.

Using these tetramers, we analyzed PBMCs from 10 individuals in whom T-cell responses to two or three of the NefYT9 peptides were detected by an ex vivo ELISpot assay. We first analyzed PBMCs from four individuals (KI-902, KI-1485, KI-1863, and KI-1915) who showed T-cell responses to both WT and 6H peptides were infected with NefQ125H virus alone or a WT/Q125H virus mixture (Figure 2f). WT-tet nonbinding, 6H-tet^+^, and 6D-tet nonbinding (WT^−^6H^+^6D^−^-tet^+^), WT^+^6H^+^6D^−^-tet^+^, and/or WT^+^6H^−^6D^−^-tet^+^ CD8^+^ T cells were found in these individuals (Table 1). All three types of T cells were found in KI-902 and KI-1863. WT^+^6H^−^6D^−^-tet^+^ and WT^−^6H^+^6D^−^-tet^+^ CD8^+^ T cells were detected in KI-1485, while WT^+^6H^+^6D^−^-tet^+^ and WT^−^6H^+^6D^−^-tet^+^ CD8^+^ T cells were found in KI-1915. These findings imply that WT-specific CD8^+^ T cells were elicited following infection with the WT virus and then NefQ125H mutant virus was selected by WT-specific T cells. After the emergence of NefQ125H mutant virus in these individuals, 6H-specific T cells and/or WT/6H cross-reactive T (CRT) cells (CRT-WT/6H) were elicited.

**Table 1:** The frequency of HLA-C*14:02-tetramer^+^ T cells in responders to NefYT9-WT, NefYT9-6H, and/or NefYT9-6D.

ID	Amino-acid at Nef125			ELISpot assay SFU/10^6^ PBMCs			Frequency of tetramer positive CD3^+^CD8^+^ cells					
	Sanger	NGS		WT	6H	6D	WT+ 6H− 6D−	WT− 6H+ 6D−	WT− 6H− 6D+	WT+ 6H+ 6D−	WT+ 6H− 6D+	WT− 6H+ 6D+
KI-902	H	N/A		1,278	1,500	65	1.67	4.93	0	0.48	0	0
KI-1485	Q/H	N/A		131	280	0	0.20	0.44	0	0	0	0
KI-1863	H	N/A		122	147	61	0.16	2.09	0	0.59	0	0
KI-1915	H	N/A		281	1,947	26	0	4.43	0	0.34	0	0
KI-1137	D	N/A		0	1,383	570	0	0.69	1.41	0	0	0
KI-1890	D	N/A		55	368	177	0	0.24	0.72	0	0	0
KI-924	H	H: 92.5%	D: 4.0%	227	845	794	0.02	0.12	0.06	0	0	0
KI-1009^a^	Q	Q: 99.0%		2,647	368	897	1.79	0.11	0.06	0.03	3.53	0
KI-1009^b^	Q/H	Q: 34.8%	H: 65.1%	N/A	N/A	N/A	3.07	1.01	0	0.28	4.94	0
KI-1205	H/D	D: 73.5%	H: 22.7%	160	140	270	0.19	0.17	1.45	0	0	0
KI-1450	H	Q: 15.8%	H: 83.3%	1,123	3,658	395	0.71	3.29	0	0	0.05	0
KI-1076	H	N/A		37	1,180	0	0	2.30	0	0	0	0
KI-1298	H	N/A		72	8,747	0	0	3.57	0	0	0	0
KI-1376	H	N/A		10	319	0	0.05	1.49	0	0	0	0
KI-1486	H	N/A		0	718	40	0.07	2.44	0.04	0	0	0
KI-1513	H	N/A		19	1,558	10	0	0.71	0	0	0	0
KI-1618	Q/H	N/A		15	156	7	0	0.17	0	0	0	0
KI-1630	H	N/A		0	2,661	0	0	4.02	0	0	0	0
KI-1491	D	N/A		76	25	3,671	0	0	2.69	0	0	0
KI-1759	D	N/A		0	60	1,656	0	0.07	1.14	0	0	0
KI-1785	D	N/A		0	78	165	0	0	0.24	0	0	0
KI-1841	D	N/A		26	71	199	0	0	0.11	0	0	0

Abbreviations: NGS, next-generation sequencing; WT, wild type; N/A, not applicable.

^a^PBMC sample collected on 2011 July 26.

^b^PBMC sample collected on 2011 September 9.

We next analyzed PBMCs from KI-1137 and KI-1890 that elicited T-cell responses to both 6H and 6D peptides and were infected with NefQ125D virus (Figure 2g). Both WT^−^6H^−^6D^+^-tet^+^ and WT^−^6H^+^6D^−^-tet^+^ CD8^+^ T cells were found in these individuals (Table 1 and Figure S1B), implying that 6H-specific T cells were elicited when this individual was infected with NefQ125H virus and then 6H-specific T cells selected NefQ125D virus. 6D-specific T cells were elicited after the emergence of NefQ125D mutant virus.

T-cell responses to all three peptides were detected in four individuals, KI-924, KI-1009, KI-1205, and KI-1450 (Figure 2h). Bulk sequence analysis showed that these individuals were infected with WT virus (KI-1009), NefQ125H virus (KI-924 and KI-1450), or a NefQ125H/NefQ125D mixture of viruses (KI-1205; Figure 2h). Deep sequence analysis confirmed the infection of WT virus alone in KI-1009 (July 2011) and the NefQ125H/NefQ125D mixture of viruses in KI-1205, whereas the NefQ125H/NefQ125D and WT/NefQ125H mixtures of viruses were detected in KI-924 and KI-1450, respectively (Table 1). Analysis using three tetramers showed that WT^+^6H^−^6D-tet^+^, WT^−^6H^+^6D^−^-tet^+^, and WT^−^6H^−^6D^+^-tet^+^CD8^+^ T cells were detected in the PBMCs of KI-924 and KI-1205 (Table 1). These results imply that WT-specific T cells, which were induced after the infection of WT virus, selected NefQ125H, and then 6H-specific T cells were elicited in these individuals. The 6H-specific T cells selected NefQ125D mutant virus and then 6D-specific T cells were elicited after the emergence of NefQ125D virus in KI-1205.

The analysis of PBMCs from KI-1450, an individual infected with the WT/NefQ125H mixture of viruses using the tetramers, demonstrated a large number of WT^−^6H^+^6D^−^-tet^+^ and WT^+^6H^−^6D^−^-tet^+^ CD8^+^ T cells and a small number of WT^+^6H^−^6D^+^-tet^+^ CD8^+^ T cells (Table 1). This result was consistent with the ELISpot assay data. NefQ125D virus was not detected by deep sequence analysis. This mutant virus might have been transiently eliminated. Deep sequence analysis confirmed that KI-1009 was infected with WT virus in July 2011 (Table S2), whereas ex vivo ELISpot assay data showed the responses to all three peptides. We investigated the existence of T cells specific for these three peptides using three tetramers. The tetramer analysis demonstrated relatively large numbers of WT^+^6H^−^6D^+^-tet^+^ and WT^+^6H^−^6D^−^-tet^+^ CD8^+^ T cells and a small number of WT^−^6H^+^6D^−^-tet^+^, WT^−^6H^−^6D^+^-tet^+^, and WT^+^6H^+^6D^−^-tet^+^ CD8^+^ T cells in this individual (Table 1 and Figure S2A), supporting the ex vivo ELISpot assay data. We next analyzed a PBMC sample collected in September 2011 using the tetramers. The results confirmed the presence of WT^+^6H^−^6D^+^-tet^+^, WT^+^6H^−^6D^−^-tet^+^, WT^−^6H^+^6D^−^-tet^+^, and WT^+^6H^+^6D^−^-tet^+^ CD8^+^ T cells, and all of these T cells were increased in abundance (Figure S2B and Table 1). Deep sequence analysis of this sample revealed a mixed infection of WT and NefQ125H viruses (Table 1). These results suggest that during a 2-month period, NefQ125H virus was selected by WT^+^6H^−^6D^−^-tet^+^, WT^+^6H^−^6D^+^-tet^+^, and/or WT^−^6H^−^6D^+^-tet^+^ CD8^+^ T cells. However, it is not clear why WT^+^6H^−^6D^+^-tet^+^ CD8^+^ T cells were elicited in this individual, even though NefQ125D virus was not detected in either KI-1450 or KI-1009. With the exception of some rare cases (i.e. KI-1450 and KI-1009), the results generally led to the hypothesis of a five-step coevolution between NefYT9-specific T cells and HIV-1.

### HLA-C*14-tetramer analysis of T cells specific for NefYT9-WT and/or mutant epitopes in the mutant virus–infected responders to only NefYT9-6H or NefYT9-6D

To clarify whether NefYT9-WT-specific CD8^+^ T cells are detected as memory T cells in the mutant virus–infected responders to only NefYT9-6H or NefYT9-6D, we analyzed six NefQ125H-infected responders to NefYT9-6H and three NefQ125D-infected responders to NefYT9-6D using three HLA-C*14:02 tetramers in addition to the analysis of KI-1298 and KI-1491 shown in Figure S1. A small number of WT^+^6H^−^6D^−^-tet^+^CD8^+^ T cells were detected in two individuals (KI-1376 and KI-1486), whereas WT^+^6H^−^6D^−^-tet^+^ CD8^+^ T cells were not found in the four NefQ125D-infected individuals (Table 1). WT^+^6H^−^6D^−^-tet^+^ CD8^+^ T cells were detected in four individuals infected with NefQ125H and/or NefQ125D (KI-902, KI-1863, KI-924, and KI-1205). These results together indicate that some individuals may be first infected with NefQ125H or NefQ125D virus, and CD8^+^ T cells specific for the NefYT9-6H or NefYT9-6D epitope are elicited in these individuals.

### Function of WT^+^6H^+^6D^−^-tet^+^ and WT^−^6H^+^6D^−^-tet^+^ CD8^+^ T cells

**Figure 3:**
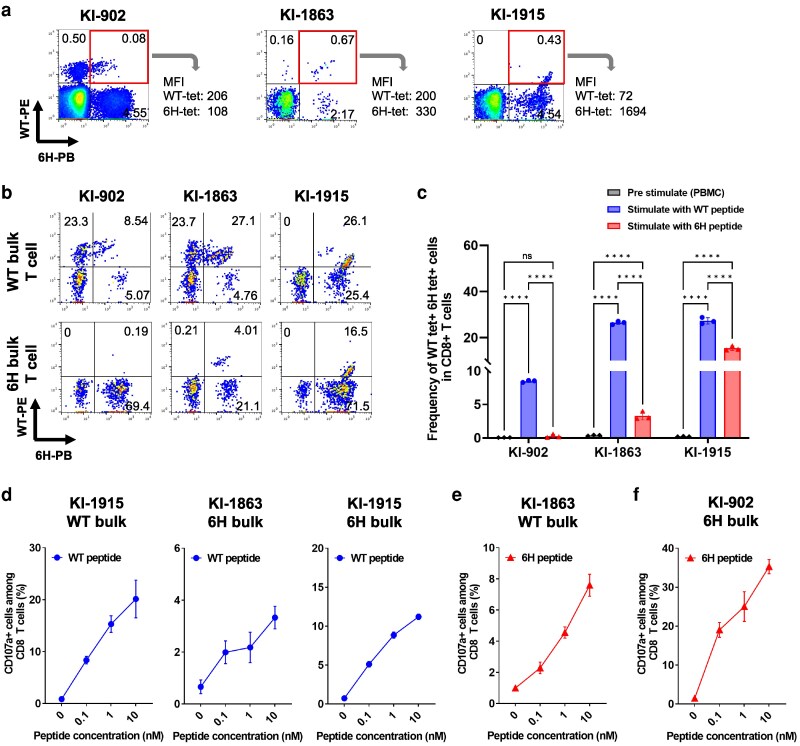
Ability of WT-tet^+^6H-tet^+^ CD8^+^ T cells to bind to HLA-C*14:02-WT and HLA-C*14:02-6H tetramers and to proliferate following stimulation with NefYT9-WT or NefYT9-6H peptide. a) Staining of PBMCs from treatment-naive HLA-C*14:02^+^ PLWH (KI-902, KI-1863, and KI-1915) with PE-conjugated HLA-C*14:02-WT-tetramer (1 nM) and PB-conjugated HLA-C*14:02-6H-tetramer (1 nM). The MFI of PE-conjugated HLA-C*14:02-WT-tetramer or PB-conjugated HLA-C*14:02-6H-tetramer in WT-tet^+^6H-tet^+^CD8^+^ T cells is presented in each figure. b) Staining of the bulk T cells cultured with NefYT9-WT (WT) or NefYT9-6H peptide (6H) with both PE-conjugated HLA-C*14:02-WT-tetramer (1 nM) and PB-conjugated HLA-C*14:02-6H-tetramer (1 nM). c) Ability of WT^+^6H^+^6D^−^-tet^+^ CD8^+^ T cells to proliferate following stimulation with NefYT9-WT or NefYT9-6H peptide. The frequency of WT^+^6H^+^6D^−^-tet^+^ CD8^+^ T cells among the total CD8^+^ T-cell population in post-stimulated T cells with NefYT9-WT or NefYT9-6H peptide was measured and compared with that in prestimulated PBMCs. The data are presented as the mean and SD (*n* = 3 biological replicates). Statistical analysis was performed using an unpaired t test (****P < 0.0001; ns, not significant). d) Expression of CD107a on NefYT9-WT-bulk and NefYT-6H-bulk T cells from KI-1863 or KI-1915 after stimulation with NefYT9-WT peptide. e) Expression of CD107a on NefYT9-WT-bulk T cells from KI-1863 after stimulation with NefYT9-6H peptide. f) Expression of CD107a on NefYT9-6H-bulk T cells from KI-902 after stimulation with NefYT9-6H peptide. d–f) Expression of CD107a on cultured bulk T cells stimulated with CD4.221-C*14:02 cells prepulsed with NefYT9-WT or NefYT9-6H peptide were tested in ICS assays (*n* = 3). The data are presented as the mean and SD.

WT^+^6H^+^6D^−^-tet^+^ CD8^+^ T cells cross-reacting to WT and NefYT9-6H epitopes were found in KI-902, KI-1863, and KI-1915, individuals who were infected with the 6H virus (Table 1). The WT^+^6H^+^6D^−^-tet^+^ CD8^+^ T cells from different individuals had different binding abilities to WT-tet or 6H-tet (Figure 3a). As these findings suggest that these T cells have different abilities to proliferate following stimulation with NefYT9-WT or NefYT9-6H peptide, we analyzed the ability of these CD8^+^ T cells to proliferate by stimulating the PBMCs of these individuals with NefYT9-WT or NefYT9-6H peptide. The frequency of WT^+^6H^+^6D^−^-tet^+^ CD8^+^ T cells was significantly increased by stimulation with NefYT9-WT peptide in all three individuals, while the frequency of WT^+^6H^+^6D^−^-tet^+^ CD8^+^ T cells was significantly increased by stimulation with NefYT9-6H peptide in only KI-1863 and KI-1915 (Figure 3b and c). The ability of WT^+^6H^+^6D^−^-tet^+^ CD8^+^ T cells to expand (i.e. the frequency of WT^+^6H^+^6D^−^-tet^+^ CD8^+^ T cells after 2 weeks of culture compared with the frequency preculture) following stimulation with NefYT9-WT peptide was greater than the ability following stimulation with NefYT9-6H peptide (Table S3). The binding ability of 6H-tet to WT^+^6H^+^6D^−^-tet^+^ CD8^+^ T cells generally correlated with the proliferative capacity of these CD8^+^ T cells.

To clarify whether WT^+^6H^+^6D^−^-tet^+^ CD8^+^ T cells have the potential to kill target cells, we analyzed the expression of degranulation marker CD107a in the cultured bulk T cells after stimulation with NefYT9-WT or NefYT9-6H peptide. WT^+^6H^+^6D^−^-tet^+^ CD8^+^ T cells were found in both NefYT9-WT-bulk and NefYT9-6H-bulk T cells derived from KI-1915 and in NefYT9-6H-bulk T cells derived from KI-1863, whereas WT^+^6H^−^6D^−^-tet^+^ CD8^+^ T cells were not present among these bulk T cells (Figure 3b). We analyzed CD107 expression on these NefYT9-WT-bulk and NefYT-6H-bulk T cells after stimulation with NefYT9-WT peptide. Approximately 20% of total CD8^+^ T cells among NefYT9-WT-bulk T cells from KI-1915 and ∼3.3 and 10% of total CD8^+^ T cells among NefYT-6H-bulk T cells from KI-1863 and KI-1915, respectively, expressed CD107a after stimulation with NefYT9-WT peptide-pulsed CD4.221-C*14:02 cells (Figures 3d and S3). These findings indicate that the majority of WT^+^6H^+^6D^−^-tet^+^ CD8^+^ T cells among these bulk T cells expressed CD107a after stimulation with NefYT9-WT peptide. We next analyzed the ability of WT^+^6H^+^6D^−^-tet^+^ CD8^+^ T cells to express CD107a after stimulation with NefYT9-6H peptide-pulsed CD4.221-C*14:02 cells. WT^+^6H^+^6D^−^-tet^+^ and WT^−^6H^+^6D^−^-tet^+^ CD8^+^ T cells were found in 27.1 and 4.76% of total CD8^+^ T cells in NefYT9-WT-bulk T cells from KI-1863 (Figure 3b). Approximately 7.6% of total CD8^+^ T cells expressed CD107a in these NefYT9-WT-bulk T cells after stimulation with NefYT9-6H peptide-pulsed CD4.221-C*14:02 cells (Figure 3e). As the frequency of WT^−^6H^+^6D^−^-tet^+^ CD8^+^ T cells is only 4.76% of total CD8^+^ T cells, at least 3% of CD8^+^ T cells that expressed CD107a were WT^+^6H^+^6D^−^-tet^+^ CD8^+^ T cells. These findings together suggest that WT^+^6H^+^6D^−^-tet^+^ CD8^+^ T cells in KI-1863 and KI-1915 have the potential to kill both NefYT9-WT peptide-pulsed and NefYT9-6H peptide-pulsed cells.

Finally, we analyzed the potential ability of WT^−^6H^+^6D^−^-tet^+^ CD8^+^ T cells to kill target cells. We only detected WT^−^6H^+^6D^−^-tet^+^ CD8^+^ T cells among NefYT9-6H-bulk T cells derived from KI-902 (Figure 3b). Approximately 35% of the total CD8^+^ T cells in NefYT9-6H bulk T cells expressed CD107a after stimulation with CD4.221-C*14:02 prepulsed with NefYT9-6H peptide (Figure 3e). These results suggest that WT^−^6H^+^6D^−^-tet^+^ CD8^+^ T cells have the potential to kill NefYT9-6H peptide-pulsed cells.

Taken together, these findings indicate that WT^+^6H^+^6D^−^-tet^+^ and WT^−^6H^+^6D^−^-tet^+^ CD8^+^ T cells are both able to kill target cells.

### Longitudinal analysis of multistep coevolution

**Figure 4:**
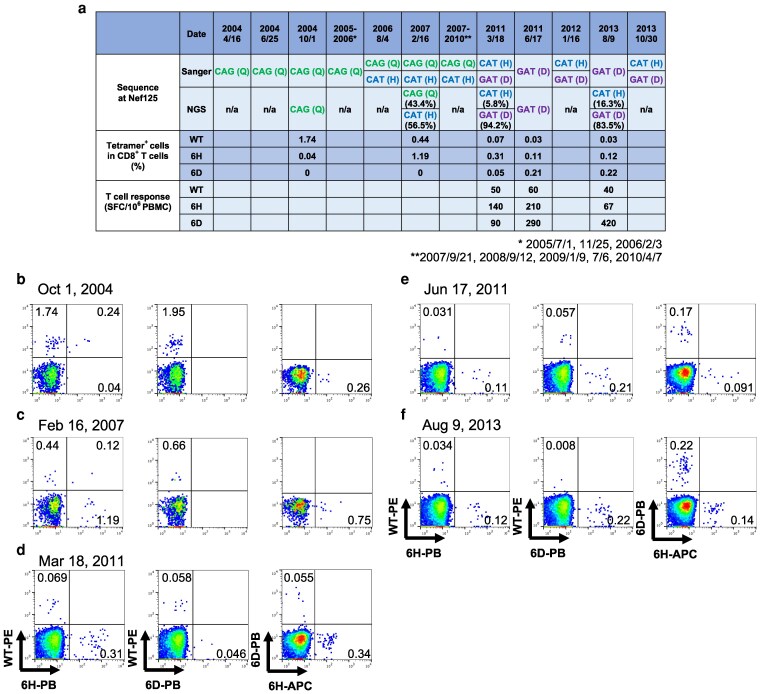
Longitudinal analysis of an HLA-C*14:02^+^ individual, KI-969, over 10 years. a) PBMC and plasma samples of a treatment-naive HLA-C*14:02^+^ PLWH, KI-969, were collected from 2004 to 2013. The sequence of position 6 of the NefYT9 epitope was analyzed by bulk-direct sequencing. The sequence was also analyzed at several time points by next-generation sequencing. The presence of NefYT9-WT-specific, NefYT9-6H-specific, and NefYT9-6D-specific HLA-C*14:02-restricted T cells was analyzed by ex vivo ELISpot assays and flow cytometric analysis using HLA-C*14:02 tetramers. The T-cell responses to each peptide at 1 µM were analyzed by ex vivo ELISPOT assays. The cutoff for a positive response was >100 spots/million PBMCs. b–f) PBMC samples were stained using the following tetramers: PE-conjugated HLA-C*14:02-WT-tetramer (1 nM), PB-conjugated HLA-C*14:02-6H-tetramer (1 nM) or APC-conjugated HLA-C*14:02-6H-tetramer (100 nM), and PB-conjugated HLA-C*14:02-6D-tetramer (1 µM). The flow cytometric analysis data of PBMCs collected on 2024 October 1 (B), 2007 February 16 (C), 2011 March 18 (D), 2011 June 17 (E), and 2013 August 9 (F), are presented.

We collected longitudinal PBMC samples from an HLA-C*14:02^+^ individual, KI-969. Bulk sequencing and deep sequence analyses showed that this individual was infected with WT virus from April 2004 to February 2006, and then with a mixture of WT and NefQ125H viruses from August 2006 to April 2010. After March 2011, this individual was infected with a mixture of NefQ125H and NefQ125D viruses or NefQ125D virus only for ∼2.5 years (Figure 4a).

We assessed T-cell responses to WT and mutant peptides by an ex vivo ELISpot assay and further analyzed the presence of HLA-C*14:02-tetramer-binding CD8^+^ T cells in PBMCs using three HLA-C*14:02-tetramers. In October 2004, WT^+^6H^−^6D^−^-tet^+^ CD8^+^ T cells were predominantly detected together with a small number of WT^+^6H^+^6D^−^-tet^+^ CD8^+^ T cells (Figure 4b), while WT^−^6H^+^6D^−^-tet^+^ CD8^+^ T cells were predominantly found in February 2007 (Figure 4c). In March 2011, when NefQ125D virus was first detected, WT^−^6H^+^6D^−^-tet^+^ CD8^+^ T cells were predominantly detected together with relatively small numbers of WT^+^6H^−^6D^−^-tet^+^ CD8^+^ T cells and WT^−^6H^−^6D^+^-tet^+^ CD8^+^ T cells (Figure 4a and d). Three months later (June 2011), the frequency of WT^−^6H^−^6D^+^-tet^+^ CD8^+^ T cells had increased (Figure 4e) and a T-cell response to NefYT9-6D peptide was detected by an ELISpot assay (Figure 4a). In August 2013, the frequency of NefYT9-6D-specific T cells was higher than that of NefYT9-6H-specific T cells, whereas NefYT9-WT-specific T cells were almost undetectable (Figure. 4a and F). These findings support the idea of a five-step coevolution between HIV-1 and NefYT9-specific T cells.

### Comparison of clinical outcomes between WT virus–, NefQ125H virus–, and NefQ125D virus–infected HLA-C*14^+^ PLWH

**Figure 5:**
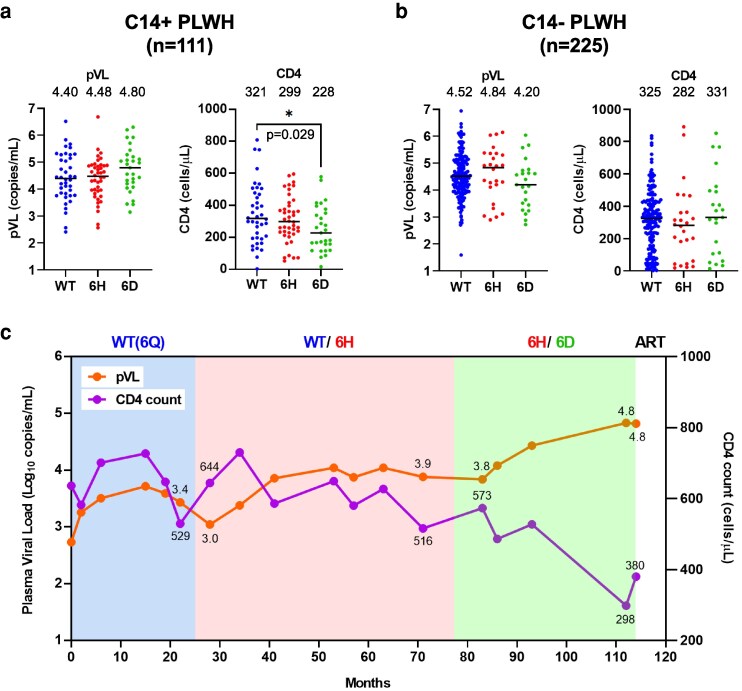
Effect of two mutations at Nef125 on the clinical outcome of treatment-naive PLWH. a and b) Comparison of the pVL and the CD4^+^ T-cell count between individuals infected with Nef125Q (WT) virus, NefQ125H (6H) virus, and NefQ125D (6D) virus among treatment-naive HLA-C*14-positive (A) or HLA-C*14-negative individuals (B). Twenty-four HLA-C*14^+^ individuals were added to 87 HLA-C14^+^ individuals infected with WT, 6H, or 6D virus, as shown in Figure 1a. The black lines and the values in each graph represent the medians of the pVL and the CD4^+^ cell count. Statistical analysis was performed using the Mann–Whitney *U* test. c) Longitudinal changes in pVL and CD4^+^ T-cell count in KI-969 (related to Figure 4a).

**Figure 6:**
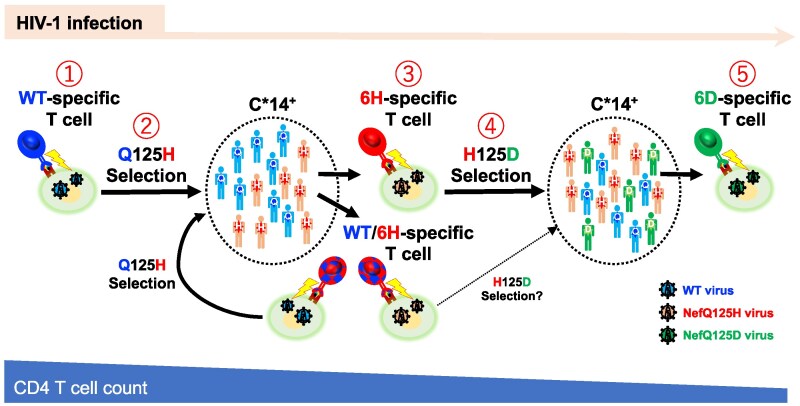
Diagram summarizing the multistep coevolution of HIV-1 and HLA-C*14-restricted T cells in HLA-C*14^+^ individuals.

We analyzed the effect of NefQ125H and NefQ125D mutations on clinical outcomes (pVL and CD4 T-cell count) in treatment-naive HLA-C*14^+^ or HLA-C*14^−^ PLWH. In 111 HLA-C*14^+^ PLWH, the CD4 T-cell count (CD4 count) in 29 NefQ125D virus–infected individuals (median: 228.0) was significantly lower than that in 40 WT-virus–infected individuals (median: 317.5; Figure 5a). The CD4 count in 42 NefQ125H virus–infected individuals (median: 298.5) was higher than that in the NefQ125D virus–infected individuals, but this difference was not statistically significant. There was no significant difference in pVL between the three groups (Figure 5a); however, the pVL in NefQ125D virus–infected individuals (median: 4.80) was generally higher than that in WT virus–infected (median: 4.40) or NefQ125H virus–infected (median: 4.48) individuals. In 225 HLA-C*14^−^ PLWH, no significant difference in CD4 count or pVL was found between the three groups (Figure 5b). In KI-969, the analysis of longitudinal samples revealed a decrease in CD4 count from 573 (March 2011) to 298 (August 2013), whereas the pVL increased from 3.8 (March 2011) to 4.8 (August 2013), indicating that clinical parameters in this individual deteriorated for ∼29 months during the stage of coinfection with NefQ125H and NefQ125D viruses or NefQ125D virus infection (Figure 6c). Taken together, these results together suggest that the accumulation of NefQ125D virus is associated with disease progression in HLA-C*14^+^ PLWH.

## Discussion

It has previously been reported that HIV-1 escape mutant-specific T cells have been elicited after the emergence of the escape mutant virus; however, the selection of a secondary escape mutation by the primary escape mutant-specific T cells has more rarely been documented (37, 38). In these cases, the primary and secondary mutations were found at different positions in the epitopes or were selected by T cells restricted by different HLA alleles. The T cells specific for the secondary escape mutation were not elicited in individuals infected with the secondary mutant virus in these previous reports. In the present study, we demonstrated that the primary and secondary mutations at the same position were selected by HLA-C*14-restricted T cells specific for the NefYT9-WT epitope and those specific for NefYT9-6H carrying the primary mutation, respectively, and that the secondary escape mutation-specific T cells were further elicited in the individuals infected with the secondary mutant virus, NefQ125D. Thus, the present study clearly exhibited that the coevolution of HIV-1 with HIV-1-specific HLA-C*14-restricted T cells progressed via five steps (Figure 6): induction of WT-specific T cells, selection of NefQ125H virus, induction of NefYT9-6H-specific T cells, selection of NefQ125D virus, and induction of NefYT9-6D-specific T cells. This pathway differs from previous reports of the multistep coevolution of HIV-1 with HLA-B*27-restricted CD8^+^ T cells (37), and HIV-1 with two different HLA-restricted CD8^+^ T cells, HLA-B*52:01-restricted and C*12:02-restricted CD8^+^ T cells (38).

Previous studies have shown that CD8^+^ T cells cross-recognizing WT and mutant HIV-1 epitopes are elicited after the emergence of escape mutant HIV-1 (11, 32, 37, 38, 40). In the present study, we observed CRT-WT/6H in three individuals infected with 6H virus (KI-902, KI-1205, and KI-1863). Interestingly, WT^+^6H^+^6D^−^-tet^+^ CD8^+^ T cells derived from KI-1205 and KI-1863 proliferated following stimulation with NefYT9-6H peptide, whereas those from KI-902 did not proliferate on stimulation. The binding ability of 6H-tet to WT^+^6H^+^6D^−^-tet^+^ CD8^+^ T cells was positively correlated with the proliferation capacity of these CD8^+^ T cells after stimulation with NefYT9-6H peptide. This finding suggests that the ability of WT^+^6H^+^6D^−^-tet^+^ CD8^+^ T cells to proliferate after stimulation with NefYT9-6H peptide is determined by TCR affinity for the HLA-C*14-NefYT9-6H complex. The TCR of CRT-WT/6H has higher affinity for the HLA-C*14-YT9-WT complex than for the HLA-C*14-YT9-6H complex, and it is therefore assumed that these CRT cells may have an increased ability to recognize WT virus-infected cells compared with mutant virus–infected cells. Analysis of CD107a expression on WT^+^6H^+^6D^−^-tet^+^ CD8^+^ T cells after stimulation with NefYT9-WT peptide or NefYT9-6H peptide demonstrated that these T cells express CD107a after stimulation with these peptides, suggesting that CRT-WT/6H may have the ability to kill both WT virus–infected and NefQ125H mutant virus–infected cells. The ability of WT^+^6H^+^6D^−^-tet^+^ CD8^+^ T cells to proliferate after stimulation with NefYT9-WT peptide was stronger than that after stimulation with NefYT-6H epitope peptide, while the expression of CD107a on WT^+^6H^+^6D^−^-tet^+^ CD8^+^ T cells after stimulation with NefYT9-WT peptide was higher than that after stimulation with NefYT9-6H peptide. These findings support the idea that CRT-WT/6H cells are capable of killing WT virus–infected cells more effectively than NefQ125H virus–infected cells. Furthermore, CRT-WT/6H cells may be capable of selecting NefQ125H virus together with NefYT9-WT-specific T cells.

The nucleotide sequences at Nef125 are CAA or CAG for Q, CAT or CAC for H, and GAT or GAC for D, suggested that Nef125 evolved from Q to H and then to D via single point mutations. The existence of Q/H and H/D mixtures support this evolutionary pathway. Analysis of T-cell responses to WT or mutant peptide in 27 HLA-C*14^+^ individuals suggested coevolution of WT to NefQ125H with NefYT9-WT-specific T cells and then NefQ125H to NefQ125D with NefYT9-6H-specific T cells. Longitudinal analysis of KI-969 confirmed this coevolutionary pathway. NefYT9-6D-specific CD8^+^ T cells were further elicited after the emergence of Nef125D virus. This is the first example of the epitope harboring the second escape mutation inducing T cells specific for the second mutation. However, it remains to be determined whether these NefYT9-6D-specific T cells can select another mutation or contribute to reverting NefQ125D virus to NefQ125H.

The dominant population of NefYT9-specific CD8^+^ T cells in KI-1009 was WT^++^6H^−^6D^−^-tet^+^ CD8^+^ T cells and WT^+^6H^−^6D^+^-tet^+^ CD8^+^ T cells. These results suggested that KI-1009 may have been infected not only with WT virus but also with NefQ125D virus. However, in this individual, NefQ125D virus was not detected at two time points and WT^−^6H^−^6D^+^-tet^+^ CD8^+^ T cells were not clearly detectable. WT^+^6H^−^6D^+^-tet^+^ CD8^+^ T cells CD8^+^ T cells may be maintained as memory T cells after KI-1009 may have first been infected with WT virus and second with NefQ125D virus. However, it is difficult to exclude the possibility that NefQ125D virus was selected by WT^−^6H^+^6D^−^-tet^+^ CD8^+^ T cells in KI-1009, and it remains unclear why NefQ125D virus could not be detected in this individual.

The present study demonstrated the multistep coevolution of HIV-1 and HIV-1-specific HLA-C*14-restricted T cells and suggests that this coevolution may lead to disease progression in HIV-1 infection. Our findings also showed that some HLA-C-restricted HIV-1-specific T cells have a strong ability to suppress HIV-1 replication and promote the multistep coevolution of HIV-1. As HLA-C-restricted HIV-1-specific T cells can effectively recognize target cells, further analysis of HLA-C-restricted T cells in other infectious diseases or cancer is expected to clarify the role of HLA-C allele-restricted T cells in the control of these diseases.

## Materials and methods

### Ethics statement

The study was approved by the ethics committees of Kumamoto University (RINRI-1340 and GENOME-342) and the National Center for Global Health and Medicine (NCGM-A-000172-01). Written informed consent was obtained from all individuals for the collection of blood and subsequent analysis according to the Declaration of Helsinki.

### Study subjects

Thirty-nine treatment-naive HLA-C*14^+^ PLWH and five treatment-naive HLA-C*14^−^ PLWH were recruited in the National Center for Global Health and Medicine. PBMCs were separated from whole blood of these PLWH and used for analysis of HLA-C*14-restricted CD8^+^ T cells specific for NefYT9, NefYT9-6H, or NefYT9-6D. HLA genotypes of the HLA-A, HLA-B, and HLA-C alleles were identified at the NPO HLA Laboratory (Kyoto, Japan).

### Cell lines

CD4.221-C*14:02 cells and CD4.221-C*14:03 cells were previously generated (41). The TAP2-deficient mouse RMA-S cell lines expressing HLA-C*14:02 (RMA-S-C*14:02) were generated, as previously described (41). These cell lines were cultured in RPMI 1640 supplemented with 10% fetal calf serum (FCS) and 0.15 mg/mL hygromycin B.

### Ex vivo interferon gamma ELISpot assay

Ex vivo interferon gamma (IFN-γ) ELISpot assay was performed, as previously described (42). First, 1 × 10^5^ PBMCs from HIV-1-infected HLA-C*14:02^+^ or HLA-C*14:03^+^ individuals and 100 nM of NefYT9, NefYT9-6H, or NefYT9-6D peptide were added to 96-well polyvinylidene plates (Millipore, Bedford, MA, USA) that had been coated overnight with 5 μg/mL anti-IFN-γ monoclonal antibody (mAb) 1-D1K (Mabtech, Nacka Strand, Sweden). Then, the plates were incubated for 16 h at 37 °C in 5% CO_2_, and plate-bound IFN-γ spots were detected, as previously described (27). The spots were counted with an Eliphoto-Counter (Minerva Tech, Kanagawa, Japan). The number of CD8^+^ T cells in PBMCs were counted by flow cytometry. The number of spots was calculated per 10^6^ CD8^+^ T cells. The cutoff for a positive response was 100 spots/10^6^ PBMCs, as described previously (27).

### HLA class I stabilization assay

The binding of peptides to HLA-C*14:02 was measured, as previously described (39). Briefly, RMA-S-C*14:02 cells were precultured at 26 °C for 16 h to fully express and present empty HLA class I molecules on the cell surface and were then incubated with 100 μM NefYT9, NefYT9-6H, and NefYT9-6D peptides at the same temperature for 1 h. The cells were then incubated at 37 °C for a further 3 h. After incubation, cells were stained with anti-HLA-C mAb DT-9 (culture supernatant of hybridoma cell line DT-9) (25) and, subsequently, with fluorescein isothiocyanate (FITC)-conjugated sheep immunoglobulin G (IgG) (Jackson ImmunoResearch Laboratories, West Grove, PA, USA). Surface expression of HLA-C*14:02 was measured by flow cytometry using the FACSCanto II (BD Biosciences, Franklin Lakes, NJ, USA). The HLA expression index was calculated as follows: (mean fluorescence intensity [MFI] of RMA-S cells prepulsed with peptide minus MFI of RMA-S cells without peptide pulsing)/(MFI of RMA-S cells maintained at 26 °C indefinitely minus MFI of RMA-S cells without peptide pulsing) ×  100.

### ICS assay

ICS assay was performed, as previously described (42). PBMCs from HIV-1-infected individuals were stimulated with NefYT9, NefYT9-6H, or NefYT9-6D peptide and cultured for 2 weeks. CD4.221-C*14:02 cells and CD4.221-C*14:03 cells prepulsed with NefYT9, NefYT9-6H, or NefYT9-6D peptide were used as stimulator cells. These cells were added to a 96-well plate together with the cultured T cells, and the cells were incubated for 4 h at 37 °C with brefeldin A (10 µg/mL). The cells were then stained with Pacific blue (PB)-labeled anti-CD3 mAb (Biolegend, San Diego, CA, USA), allophycocyanin (APC)-labeled anti-CD8 mAb (Biolegend), and LIVE/DEAD Fixable Near-IR Dead Cell Stain Kit (Invitrogen, Carlsbad, CA, USA), and subsequently fixed with 4% paraformaldehyde and incubated in permeabilization buffer (0.1% saponin–5% FCS–phosphate-buffered saline). Thereafter, the cells were stained with phycoerythrin (PE)-labeled anti-IFN-γ mAb (Biolegend). Staining data were acquired using the FACSCanto II (BD Biosciences) and analyzed using FlowJo 10.7.1 software.

### Degranulation assay

PBMCs from HIV-1-infected individuals, KI-902, KI-1863, and KI-1915 were stimulated with each NefYT9 or NefYT9-6H peptide and cultured for 2 weeks. CD4.221-C*14:02 cells prepulsed with NefYT9 or NefYT9-6H peptide were used as stimulator cells. These cells were added to a 96-well plate together with the cultured T cells, and the cells were incubated for 4 h at 37 °C with APC-labeled anti-CD107a (LAMP-1) mAb (Biolegend). The cells were then stained with PB-labeled anti-CD3 mAb (Biolegend), PerCP-Cyanine5.5-labeled anti-CD8 mAb (Biolegend), and LIVE/DEAD Fixable Near-IR Dead Cell Stain Kit (Invitrogen). Staining data were acquired using the FACSCanto II (BD Biosciences) and analyzed using FlowJo 10.7.1 software.

### Bulk sequencing and deep sequencing of HIV-1 NefYT9

Bulk sequencing of the HIV-1 NefYT9 encoding region (Nef120-128) of plasma viral RNA from 26 HLA-C*14:02^+^ or HLA-C*14:03^+^ individuals infected with HIV-1, as well as longitudinal samples from one individual, was performed. Plasma was separated from whole blood and stored at −80 °C. HIV-1 viral RNA was extracted from plasma samples using a QIAamp UltraSens virus kit (Qiagen, Venlo, The Netherlands) and was then subjected to two rounds of PCR. PCR products and sequences were analyzed, as previously described (18). Several PCR products for bulk sequencing were used for deep sequencing. The preparation of libraries and deep sequencing were performed by FASMAC (https://fasmac.co.jp/en; Kanagawa, Japan) using MiSeq (Illumina, San Diego, CA, USA).

### Nucleotide sequence accession numbers

The accession numbers of previously determined sequences used in this study are AB873602 to AB873907 (18).

### Tetramer staining of HLA-C*14-restricted T cells specific for NefYT9 or its mutant peptide

HLA class I-peptide tetrameric complexes (tetramers) were synthesized, as described previously (43–45). NefYT9 (WT), NefYT9-6H (6H), or NefYT9-6D (6D) peptides were added to the refolding solution containing the biotinylation sequence–tagged extracellular domain of the HLA-C*14:02 molecule and β_2_-microglobulin. The purified monomer complexes were mixed with PE- or APC-labeled streptavidin (Invitrogen) or PB-labeled streptavidin (Biolegend). Tetramer staining of T cells was performed as previously described (42). PBMCs or cultured T cells were stained with the tetramers at 37 °C for 30 min. The cells were then washed twice with R10, followed by staining with FITC-labeled anti-CD3 mAb (Biolegend), PerCP-Cyanine5.5-labeled anti-CD8 mAb (Biolegend), and LIVE/DEAD Fixable Near-IR Dead Cell Stain Kit (Invitrogen) at 4 °C for 30 min. The cells were washed twice with R10. Data were analyzed using the FACSCanto II (BD Biosciences), and FlowJo 10.7.1 software.

To evaluate the background level of staining for the samples treated with each tetramer, PBMCs from five treatment-naive HLA-C*14^−^ PLWH were stained with WT-tetramer (PE) and 6H-tetramer (PB), with WT-tetramer (PE) and 6D-tetramer (PB), or with 6H-tetramer (APC) and 6D-tetramer (PB). The mean ± 3SD values for the frequency of tetramer^+^ CD8^+^ T cells were 0 and 0.085 in the WT (PE) and 6H (PB), 0 and 0.026 in the WT (PE) and 6D (PB), and 0.013 and 0.050 in 6H (APC) and 6D (PB), respectively. The frequencies of tetramer^+^ CD8^+^ T cells in HLA-C*14^+^ PLWH shown in Table 1 were corrected by subtracting these values.

## Supplementary Material

pgag105_Supplementary_Data

## Data Availability

The datasets of 306 previously determined HIV-1 sequences used in this study were reported in AB873602 to AB873907 (18). The datasets analyzed during the current study are presented in all figures and a table and in the Supplementary material.
