# Supplementary material for: Multistep coevolution of HIV-1 and human leukocyte antigen-C-restricted HIV-1-specific CD8+ T cells and the association with disease progression
Source: PNAS Nexus. 2026 Apr 7;5(4):pgag105. doi: 10.1093/pnasnexus/pgag105 (PMC13096738; doi:10.1093/pnasnexus/pgag105)
Supplement: pgag105_Supplementary_Data [file pgag105_supplementary_data.pdf]

Supplementary Figure 1.

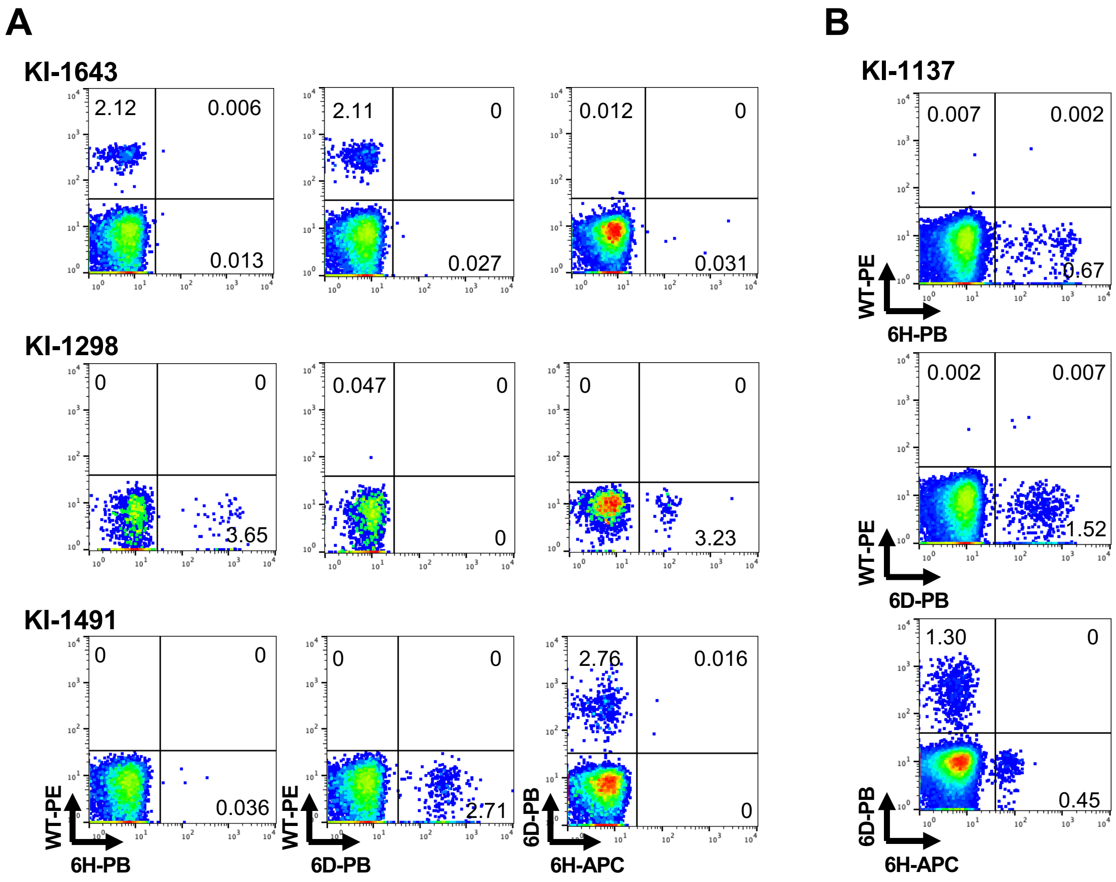

Supplementary Figure 1. Staining of PBMCs from KI-1643, KI-1298, and KI-1491 (who responded to a single peptide) and KI-1137 with three HLA-C*14:02 tetramers (related to Fig. 2E–H).

PBMCs from KI-1643, KI-1298, KI-1491 (**A**), and KI-1137 (**B**) were analyzed using the following combinations of tetramers: PE-conjugated HLA-C*14:02-WT-tetramer (1 nM) and PB-conjugated HLA-C*14:02-6H-tetramer (1 nM), PE-conjugated HLA-C*14:02-WT-tetramer (1 nM) and PB-conjugated HLA-C*14:02-6D-tetramer (1 nM), and APC-conjugated HLA-C*14:02-6H-tetramer (100 nM) and PB-conjugated HLA-C*14:02-6D-tetramer (1 nM). These stained PBMCs were analyzed by flow cytometry.

Supplementary Figure 2.

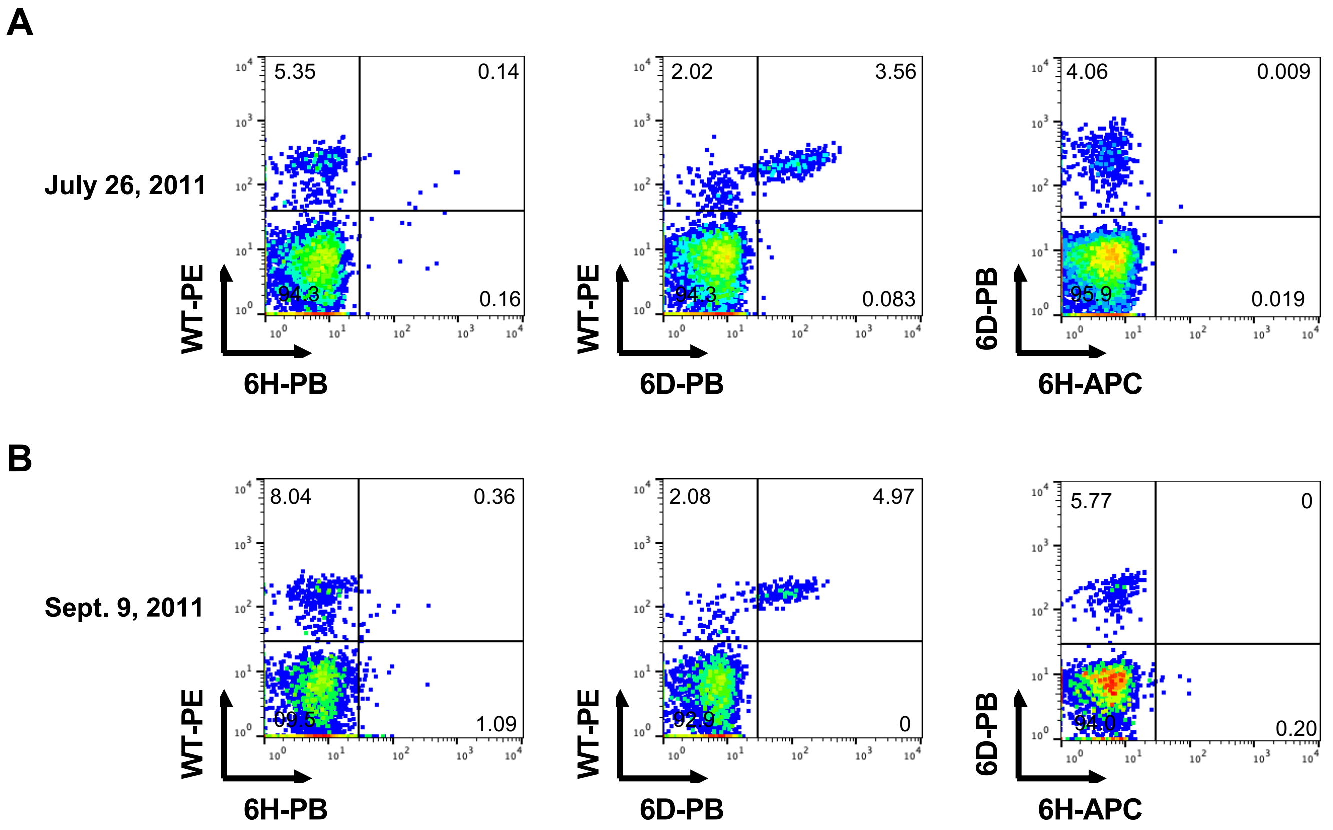

Supplementary Figure 2. Identification of CD8+ T cells specific for NefYT9-WT and mutant peptides in a treatment-naïve HLA-C*14+ PLWH, KI-1009, using HLA-C*14:02-tetramers. PBMCs from a HLA-C*14:02+ individual, KI-1009, were analyzed using the following combinations of tetramers: PE-conjugated HLA-C*14:02-YT9-tetramer (1 nM) and PB-conjugated HLA-C*14:02-6H-tetramer (1 nM), PE-conjugated HLA-C*14:02-YT9-tetramer (1 nM) and PB-conjugated HLA-C*14:02-6D-tetramer (1 nM), or APC-conjugated HLA-C*14:02-6H-tetramer (100 nM) and PB-conjugated HLA-C*14:02-6D-tetramer (1 nM). The PBMCs of KI-1009 collected on July 26, 2011 (A) and on September 9, 2011 (B) were analyzed by flow cytometry.

Supplementary Figure 3.

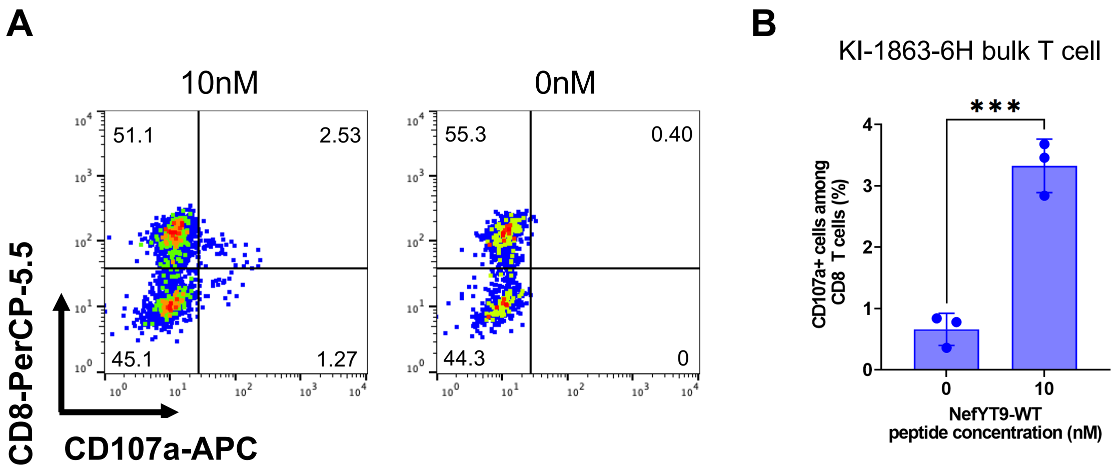

Supplementary Figure 3. CD107a expression of NefYT9-6H-bulk T cells in KI-1863.

A. A representative case of CD107a expression of NefYT9-6H-bulk T cells in KI-1863, as shown in Fig. 3D. **B**. The mean and SD of the frequency of CD107a+CD8+ cells among total CD8+ T cells in NefYT9-6H-bulk T cells stimulated with NefYT9-WT peptide at 10 nM are presented. Triplicate samples were analyzed. Statistical analysis was performed using an unpaired t-test (***, $P < 0.001$).

Supplementary Table 1.

Supplementary Table 1. HLA-C*14-associated mutations in NefYT9, NefIF8, and GagLY9 epitopes.

HLA allele	Gene	Epitope	Position	Target Amino Acids (AA)	2x2 contingency table*				**p-value
					HLA+ AA+	HLA+ AA-	HLA- AA+	HLA- AA-	
HLA-C*14	Nef	NefIF8 NefYT9	120	F	54	26	92	142	0.000016
HLA-C*14	Nef	NefYT9	125	D	19	69	23	218	0.0053
HLA-C*14	Nef	NefYT9	125	H	28	60	22	219	0.0000016
HLA-C*14	Gag	GagLY9	81	A	52	55	72	218	0.0000081

*HLA+AA+; # of patient with both HLA and amino acid. HLA+AA-; # of patients with HLA and without the amino acid. HLA-AA+; # of patients without HLA and with the amino acid. HLA-AA-; # of patients without HLA and without the amino acid.
**p-value was calculated by Fisher's exact test.

Supplementary Table 2.

Supplementary Table 2. Frequency of codon variation of Nef125 analyzed by NGS in the plasma sample from KI-1009 (July 26, 2011).

	Reads	CAA (Q)	CAG (Q)
Nef125	271510	99.7%	0.2%

Supplementary Table 3.

Supplementary Table 3. Frequency of both WT-tetramer and 6H-tetramer positive T cells in PBMC and bulk T cells that were established by stimulation of WT peptide or 6H peptide.

.

	Frequency of WT-tet+ 6H-tet+ T cells							
	Pre-stimulation (PBMC)	SD	Stim with WT	SD	Ratio to Pre	Stim with 6H	SD	Ratio to Pre
KI-902	0.08	0.00	8.5	0.1	111.5	0.3	0.2	3.8
KI-1863	0.40	0.06	26.5	0.6	66.7	3.3	0.5	8.4
KI-1915	0.23	0.03	27.3	1.1	117.0	15.4	0.8	66.0
